# Molecular Chaperone BRICHOS Inhibits CADASIL-Mutated NOTCH3 Aggregation *In Vitro*

**DOI:** 10.3389/fmolb.2022.812808

**Published:** 2022-02-09

**Authors:** Daniel V. Oliveira, Julia Svensson, Xueying Zhong, Henrik Biverstål, Gefei Chen, Helena Karlström

**Affiliations:** ^1^ Department of Neurobiology, Care Sciences and Society, Karolinska Institutet, Stockholm, Sweden; ^2^ Department of Biomedical Engineering and Health Systems, School of Engineering Sciences in Chemistry, Biotechnology and Health, KTH Royal Institute of Technology, Huddinge, Sweden; ^3^ Department of Biosciences and Nutrition, Karolinska Institutet, Huddinge, Sweden

**Keywords:** BRICHOS inhibits NOTCH3 aggregation, CADASIL, Notch3, misfolded protein, BRICHOS, VSMC = vascular SMC

## Abstract

CADASIL (cerebral autosomal dominant arteriopathy with subcortical infarcts and leukoencephalopathy) is the most common familial form of stroke, which is caused by mutations located in the epidermal growth factor (EGF)-like repeats of the *NOTCH3* gene. Mutations cause the NOTCH3 (N3) protein to misfold and aggregate. These aggregates will be a component of granular osmiophilic material, which when accumulated around the arteries and arterioles is believed to cause the degradation of vascular smooth muscle cells (VSMC). VSMC degradation affects blood flow regulation and leads to white matter and neuronal death. Currently, there is no treatment for CADASIL. The dementia-relevant BRICHOS domain is a small multitalented protein with functions that include ATP-independent chaperone-like properties. BRICHOS has been shown to prevent the aggregation of both fibrillar and non-fibrillar structures. Therefore, the objective of this study is to investigate whether BRICHOS exhibits anti-aggregating properties on a recombinant CADASIL-mutated N3 protein consisting of the first five repeats of EGF (EGF_1–5_), harboring a cysteine instead of an arginine in the position 133, (R133C). We found that the N3 EGF_1–5_ R133C mutant is more prone to aggregate, while the wildtype is more stable. Recombinant human Bri2 BRICHOS is able to interact and stabilize the R133C-mutated N3 protein in a dose-dependent manner. These results suggest an anti-aggregating impact of BRICHOS on the N3 EGF_1–5_ R133C protein, which could be a potential treatment for CADASIL.

## Introduction

Small-vessel diseases (SVD) are characterized by pathological changes in small- and medium-sized vessels in the body and are among the leading causes of stroke. The most well-known hereditary form of SVD is cerebral autosomal dominant arteriopathy with subcortical infarcts and leukoencephalopathy (CADASIL), which is caused by mutations in the *NOTCH3* gene (Joutel et al., 1996; Pantoni, 2010). The disease usually manifests with migraines with aura around the age of 30 years and progresses with more severe symptoms, in which the individual can experience recurrent strokes, cognitive decline, and dementia due to damage to white matter (Joutel et al., 1996; Joutel et al., 2001; Chabriat et al., 2009). The mean life expectancy of the CADASIL patient is only 64.6 years for men and 70.7 years for women (Opherk et al., 2004). It affects approximately 5/100,000, which probably is an underestimation (Razvi et al., 2005; Narayan et al., 2012; Moreton et al., 2014; Rutten et al., 2016). Currently, there are no therapies to prevent CADASIL.

*The NOTCH3* gene encodes a type-1 transmembrane receptor, NOTCH3 (N3), which is expressed mainly in pericytes and vascular smooth muscle cells (VSMC). Notch signaling is essential during embryogenesis, cell fate determination processes, and vascular biology (Krebs et al., 2003; Domenga et al., 2004; Andersson et al., 2011). The cell surface receptor is 300 kDa and includes a large extracellular domain (ECD) with 34 epidermal growth factor (EGF)-like repeats, a transmembrane spanning sequence, and an intracellular domain (ICD) (Andersson et al., 2011).

Most of all, CADASIL-causing mutations are located in the EGF-like repeats in the N3 ECD. Each EGF-like repeat contains six cysteine (Cys) residues, which are important for stabilizing the domain by forming three disulfide bridges. All mutations result in a loss or a gain of a Cys residue in one of the EGF-like repeats. Consequently, there will be an odd number of Cys residues, leaving one of the Cys residues unpaired. This will likely lead to folding problems and a changed conformation of the N3 ECD (Rutten et al., 2014). This is believed to lead to the accumulation and deposition of amorphous (non-fibrillar) N3 ECD on the cell surface of the VSMC (Joutel et al., 2000; Monet-Lepretre et al., 2013), which are a component of the granular osmiophilic material (GOM) (Joutel et al., 2010). This accumulation of GOM surrounding the VSMC is believed to be a causative effect of the degeneration of the VSMC. VSMC degeneration is prominent in the brain and alters blood flow regulation, leading to white matter and neuronal loss, vessel fibrosis, and lumen stenosis (Ruchoux et al., 1995; Pfefferkorn et al., 2001).

There are several ideas on how to remove these aggregates by using immunotherapy, enhancing degradation pathways, such as autophagy or ubiquitin, or inhibiting the aggregation by chemical compounds or protein chaperones.

Many neurodegenerative diseases are caused by protein misfolding and aggregation, i.e., both non-fibrillar amorphous structures and fibrillar amyloid aggregates. Therefore, molecular chaperones are important in promoting proteins to fold accurately (Balchin et al., 2016). The BRICHOS protein is a recently established functional domain with approximately 100 amino acids in size, found in more than 1,000 proteins (Hedlund et al., 2009). It is a multitalented domain with functions including chaperone-like properties and is proposed to assist proteins during folding (Sanchez-Pulido et al., 2002). There are 12 families of BRICHOS-containing proproteins; among them, Bri2, also called integral transmembrane protein 2B (ITM2B), is expressed in the central nervous system (CNS) (Hedlund et al., 2009; Poska et al., 2016).

Research related to amyloid-β (Aβ) peptide in Alzheimer’s disease (AD) unraveled that the different quaternary structures of the Bri2 BRICHOS domain can exhibit various functions. Bri2 BRICHOS monomers and dimers can interact with aggregates and reduce and counteract Aβ42-induced neurotoxicity and Aβ42 fibrillization, respectively (Chen et al., 2017). Bri2 BRICHOS oligomers can inhibit non-fibrillar aggregations of citrate synthase. It is theorized that the oligomers can act on these aggregates since the BRICHOS protein has a hydrophobic region. Thus, oligomers will have larger and/or more hydrophobic regions exposed, making them sticky to non-fibrillar structures with more hydrophobic regions on the surface. However, how BRICHOS interact with aggregates at the molecular level remains unknown (Chen et al., 2017). As an efficient molecular chaperone, BRICHOS could potentially interact with the N3 protein and prevent aggregation, thus delaying the progression of CADASIL. In this report, we have explored the Notch3 multimerization/aggregation process with a common and well-established CADASIL mutation (R133C) and whether this process can be prevented or reduced in the presence of the Bri2 BRICHOS chaperone. We have found that Bri2 BRICHOS can stabilize the mutant Notch3 protein in a monomeric form visualized by native gel and transmission electron microscopy. Furthermore, using a turbidity assay, we also observed that BRICHOS reduced the aggregation kinetics of the Notch3 mutant by 50% in a 1:1 molar ratio. These results are very encouraging, and the next step will be to test this strategy *in vivo* in a CADASIL mouse model.

## Materials and Methods

### Generation of Stable HEK293 NOTCH3 EGF_1–5_ WT and R133C Cell Lines

We used previously generated constructs encoding a truncated form of human Notch3 ECD [wildtype (WT) and R133C] consisting of the first five EGF-like repeats (N3 EGF_1–5_, amino acids 1–234) with poly-histidine and c-Myc tags at the C-terminus (Duering et al., 2011). From these original constructs, we generated codon-optimized constructs using GeneArt gene synthesis (ThermoFisher) in the *piggyBac* transposon system to increase the yield of secreted protein. Human embryonic kidney 293 (HEK293) cells were seeded in six-well plates (0.5 × 10^6^ cells per well) and cultured in Dulbecco's modified Eagle medium (DMEM; Invitrogen) supplemented with 10% fetal bovine serum (FBS; Invitrogen) and 1% penicillin-streptomycin (Invitrogen) at 37°C in a humidified 5% CO_2_. The following day, 0.5 µg of N3 EGF_1–5_ WT and R133C plasmids containing the *piggyBac* transposon and 0.2 µg of *piggyBac* transposase were transfected using Lipofectamine 2000 (Invitrogen) according to the manufacturer’s instructions. Twenty-four hours after transfection, cells were split and selected for 2 weeks in DMEM medium containing 1 mg/ml of G418 (Invitrogen). After 2 weeks, the selected cells were expanded and maintained in DMEM medium with 0.6 mg/ml of G418.

### Purification of NOTCH3 EGF_1–5_ WT and R133C Proteins

HEK293 N3 EGF_1–5_ WT and R133C cells (N3 EGF_1–5_ WT and R133C) cells were grown in DMEM with 10% FBS in a 225 cm^2^ flask until near confluence and were then washed with Dulbecco’s phosphate buffered saline (DPBS) and supplemented with DMEM medium without FBS for 5 days. The conditioned medium of both cell lines was collected, cleared of cell debris by centrifugation at 1,500 *g*, and dialyzed with the aid of SnakeSkin dialysis tubing 10 kDa MWCO (ThermoFisher) in PBS for 24 h at 4°C. Dialyzed media from three flasks (∼150 ml) of each cell line was equilibrated with 1 ml (bead volume) of cobalt ion affinity resin (TALON Superflow, GElifesciences) for 30 min with agitation at 4°C. Then the resin solution was added on a gravity flow column and washed three times with wash buffer (200 mM sodium phosphate buffer, 500 mM NaCl and 5 mM imidazole, at pH 7.5). After elution with elution buffer (200 mM sodium phosphate buffer, 500 mM NaCl and 300 mM imidazole, at pH 7.5), fractions were pooled and dialyzed in a dialysis membrane (Spectrum™ Spectra/Por™ 1 6–8 kDa MWCO, FisherScientific) against PBS for 24 h at 4°C. The pooled fractions of N3 EGF_1–5_ WT and R133C were concentrated at 1 mg/ml in a concentration column (Vivaspin 6 10 kDa MWCO, Sartorius) according to the manufacturer’s instructions.

### Bri2 BRICHOS Domain Purification

NT-Bri2 BRICHOS shuffle *Escherichia coli* cells were cultured at 30°C in Luria–Bertani (LB) medium containing 15 μg/ml kanamycin as previously described (23). Cells were induced by 0.5 mM isopropyl β-d-1-thiogalactopyranoside (IPTG) after OD600 reached ∼0.9 at 20°C. Induced cells were harvested (5,000 rpm, 20 min, 4°C) and resuspended with 20 mM Tris pH 8.0 and stored at −20°C. NT-Bris2 cells were thawed, sonicated (2 s on, 2 s off, 65% of the maximum amplitude), and centrifuged at 24,000 *g* to separate cell remnant and the desired proteins. The supernatant was poured into the Ni-NTA-column for affinity chromatography and to allow the His-tagged protein to bind to the nickel in the beads. The beads were washed with 20 mM Tris-HCl pH 8.0 with 20 mM imidazole to remove unspecific proteins and bindings. The NT-Bri2 BRICHOS protein was eluted with 300 mM imidazole in 20 mM Tris-HCl pH 8.0, and the elution was dialyzed in a cold room overnight in 20 mM Tris pH 8.0 using a Spectra/Por 6-8kD RC membrane. Next, thrombin (1:1,000, w/w) was added to the proteins to cleave the NT tag from Bri2 BRICHOS. The protein solution was re-run in the Ni-NTA-column, of which the NT tag bound to the beads and the flow-through containing the Bri2 BRICHOS domain was collected.

### Polyacrylamide Gel Electrophoresis by Sodium Dodecyl Sulfate Polyacrylamide Gel Electrophoresis

Sodium dodecyl sulfate (SDS) loading buffer and dithiothreitol (DTT) were added to the samples (purified proteins of N3 EGF_1–5_ WT and R133C and Bri2 BRICHOS) and heated at 95°C for 5 min for sodium dodecyl sulfate polyacrylamide gel electrophoresis (SDS-PAGE) under reducing conditions. For non-reducing SDS-PAGE, the same samples were combined with SDS loading buffer that did not include DTT and processed as described above. Samples were separated on 4–20% gradient gels and stained with Coomassie Brilliant blue.

### Tertiary Structure Modelling

The tertiary structure models of N3 EGF_1–5_ were modelled by the I-TASSER server (https://zhanglab.ccmb.med.umich.edu/I-TASSER/) (Roy et al., 2010), and the models with the best C-scores (−0.23 for WT and −2.14 for R133C) were selected and visualized with UCSF Chimera (Pettersen et al., 2004).

### Circular Dichroism Spectrometer

Circular dichroism spectra were recorded in quartz cuvettes of 1 mm path length from 260 to 185 nm on a J-1500 Circular Dichroism Spectrophotometer (JASCO, Japan) with a protein concentration of 8 µM. Spectra were collected from 25°C to 125°C with 10°C as an interval. The wavelength step was 0.5 nm, averaging time 0.3 s, time constant 100 ms, and bandwidth 1 nm. During all the measurements, the HT voltage was lower than 600 V. The spectra shown are averages of three consecutive scans.

### Co-Incubation of WT N3 EGF_1–5_ and R133C Mutant With BRICHOS

The WT N3 EGF_1–5_ and R133C mutants were diluted with 1 × PBS to 30 µM and then equilibrated at 37°C with and without recombinant Bri2 BRICHOS at 1:1 and 1:2 molar for 80 h. The final products were combined with native loading buffer, free of DTT and SDS, and separated on a 6% native PAGE gel.

### Turbidity Assay

The WT N3 EGF_1–5_ and R133C mutants were diluted with 1 × PBS to 10 µM and then equilibrated at 37°C with and without recombinant Bri2 BRICHOS in 1:1 ratio. The aggregation kinetics was measured by reading the apparent increase in absorbance at 360 nm using a microplate reader (FLUOStar Galaxy from BMG Labtech, Offenberg, Germany) with shaking (240 s 300 rpm before each cycle; the interval for each cycle was 5 min).

### Negative-Stain Preparation and Transmission Electron Microscopy Imaging

Aliquots (4 μl) of the samples after 80 h of incubation with and without Bri2 BRICHOS were adsorbed onto glow-discharged continuous carbon-coated copper grids (400 mesh, Analytical Standards) for 2 min. The excessive sample solution was blotted with a filter paper piece, subsequently washed with two drops of Milli-Q water, and negatively stained with one drop of 2% (w/v) uranyl acetate for 45 s before final blotting and air drying. The samples were imaged using a JEOL JEM2100F field emission gun transmission electron microscope (JEOL, Japan) operating at 200 kV. Single micrographs of the sample were recorded on a TemCam-XF416® camera, TVIPS (Tietz Video and Image Processing Systems, GmbH, Gauting, Germany) at a nominal magnification of 50,000 and 0.5–2.5 μm defocus.

## Results

### Generation and Characterization of WT and R133C Mutant N3 EGF_1–5_ Proteins

**FIGURE 1:**
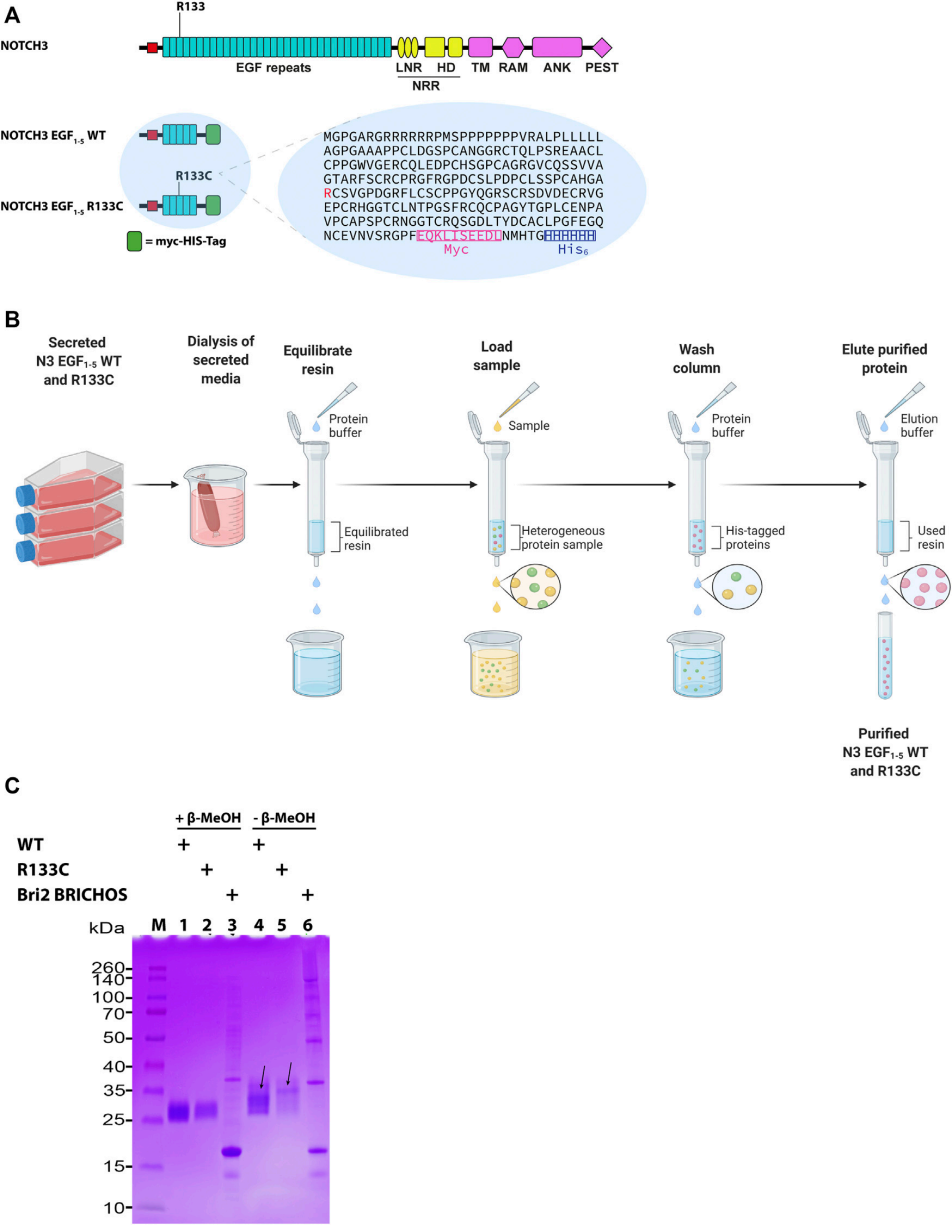
The architecture of N3 EGF_1–5_. **(A)** Schematic representation of NOTCH3 and NOTCH3 EGF_1–5_ WT and R133C. NOTCH3 represents the full-length protein, and NOTCH3 EGF_1–5_ represents the NOTCH3 protein with exons 1 to 5 fused with a myc-His-tag at the C-terminus used for purification of proteins. Arg133, which is mutated to CysR133, is shown in red in the amino acid sequence. **(B)** Schematic representation of the purification of the N3 EGF_1–5_ WT and R133C proteins. **(C)** SDS-PAGE of purified WT N3 EGF_1–5_ and R133C mutant proteins under reducing and non-reducing conditions. In addition, the recombinant Bri2 BRICHOS was also analyzed in a similar way. The arrows depict the plausible different monomeric forms with different molecular intramolecular disulfide bonds under non-reduced condition. The SDS-PAGE is a representative figure of three independent experiments performed in triplicate (*n* = 3).

The Notch3 receptor is composed of 34 EGF repeats in the ECD, and the R133C mutation is located in the EGF_3_ repeat and thus lies within the first five EGF domains in the recombinantly produced EGF_1–5_ proteins (Figure 1A). Both WT N3 EGF_1–5_ and the R133C mutant were expressed in human HEK 293 cells and purified by immobilized metal affinity chromatography (Figure 1B). The final purified proteins of both WT and the R133C showed excellent purity as detected by SDS-PAGE (Figure 1C). To find out whether the recombinant WT N3 EGF_1–5_ and the R133C mutant form disulfide bridge-dependent oligomers, we analyzed the recombinant proteins under reducing and non-reducing conditions by SDS-PAGE (Figure 1C). Under reducing conditions, only one band appeared for both WT N3 EGF_1–5_ and the R133C mutant with a size between 25 and 35 kDa indicated by the protein marker. Under non-reducing conditions, the majority of the WT protein was still within a monomer size, indicating there was no disulfide bridge-dependent oligomer formation. However, several weaker bands were present under non-reducing conditions, which migrated slower compared to reducing conditions, suggesting that intramolecular disulfide bonds were formed within the WT Notch3 protein. Similarly, the R133C mutant showed a similar phenomenon in which it migrated slower under non-reducing conditions than under reducing conditions. However, unlike the WT, the mutant mainly showed one band under non-reducing conditions, while the WT migrated with four distinguishable bands, indicating that the arginine (Arg) to Cys mutation can alter the pattern of intramolecular disulfide bridge formation.

### Prediction of the Structure of the N3 EGF_1–5_ Proteins

**FIGURE 2:**
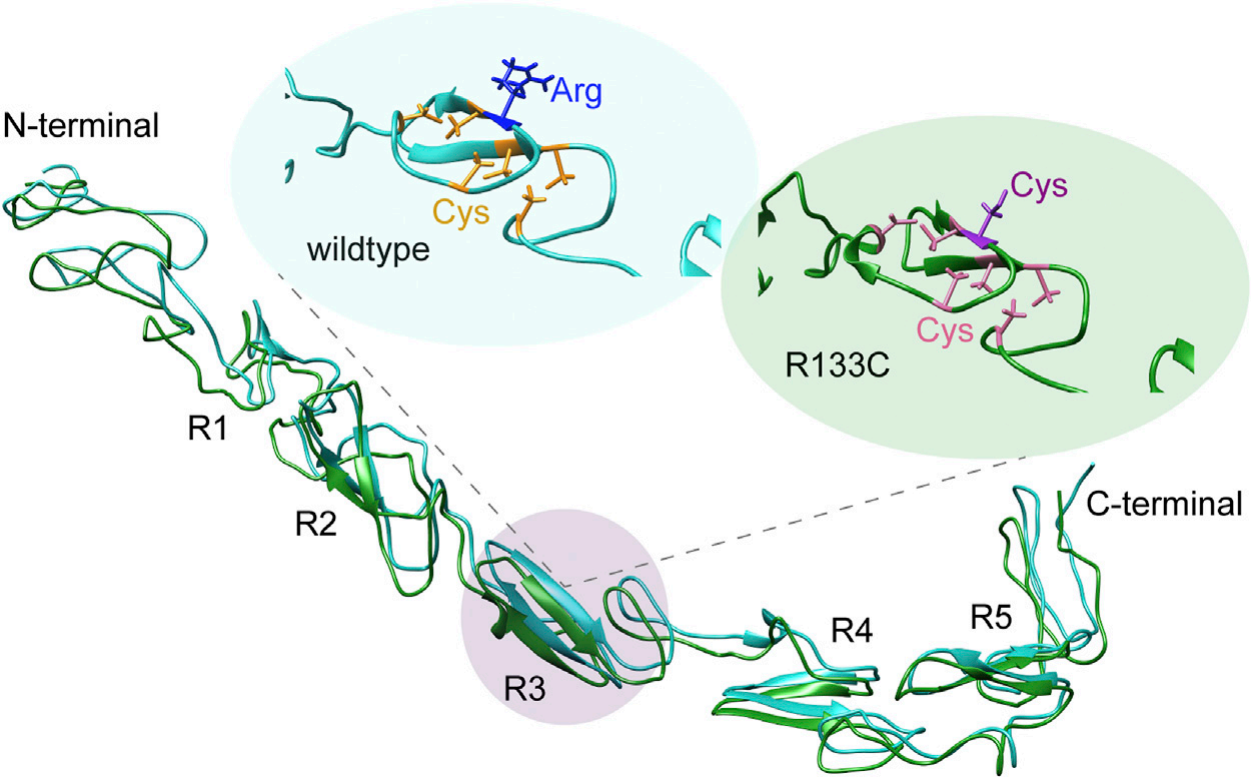
Structural analysis of N3 EGF_1–5_. **(A)** Tertiary structure models of the wildtype N3 EGF_1–5_ and R133C mutant modelled by I-TASSER, while C-scores of the modelling are −0.23 and −2.14, respectively. EGF repeats are labeled with R1, R2, R3, R4, and R5. The Cys residues in the WT N3 EGF_1–5_ and R133C mutant are shown in yellow **(B)** and pink **(C)**, respectively. The position of wildtype R133 and mutation C133 is in blue and purple, respectively.

The complete N3 EGF structure is still not available; however, there is a crystal structure of Notch1 (EGF_8–12_) bound to Jagged1 (N-EGF_3_) (Luca et al., 2017). Here, we modelled the WT N3 EGF_1–5_ and the R133C mutant with the I-TASSER method. The tertiary structure model of WT N3 EGF_1–5_ showed a linear structure consisting of five repeats containing the β-hairpin structure, i.e., R1, R2, R3, R4, and R5 (Figure 2A). With the R133C mutation, the overall structure was quite identical to the WT counterpart; both are largely unstructured (Figure 2A), suggesting that the R133C mutation does not change the overall tertiary structure of EGF_1–5_. Furthermore, in the R3 region, there are three pairs of Cys residues in WT EGF_1–5_, which are highly possible to form three disulfide bridges according to spatial locations. Arg133 is solvent-exposed (Figure 2B). In the EGF_1–5_ R133C mutant, similarly, there are three pairs of Cys residues; however, the unpaired Cys133 points outside, is solvent-exposed (Figure 2C), and could be active to affect the intramolecular disulfide bridges.

### Secondary Structure Analysis of N3 EGF_1–5_ Proteins

**FIGURE 3:**
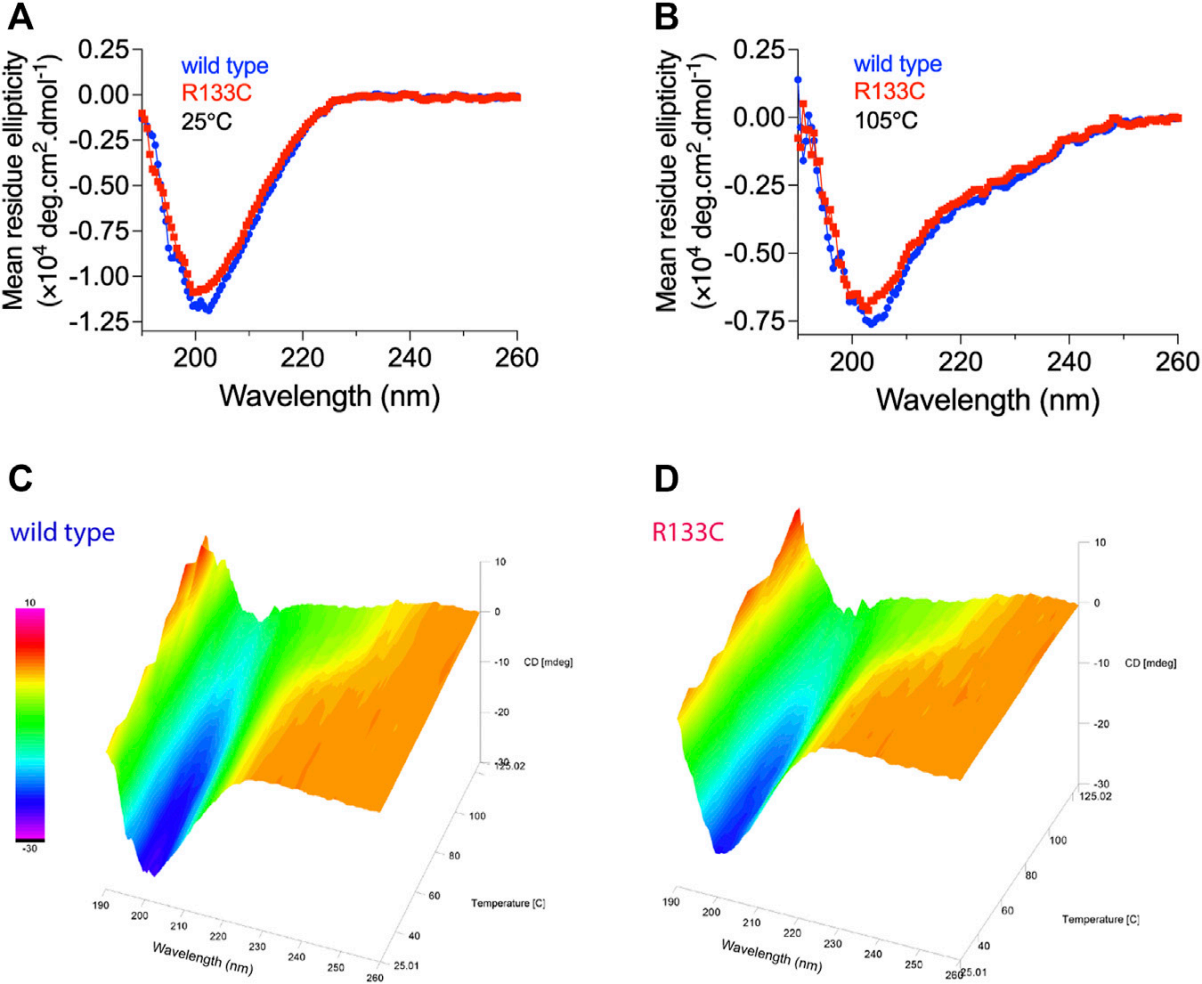
Structural characterization of WT N3 EGF_1–5_ and R133C mutant. CD spectrum measurements of **(A)** wildtype N3 EGF_1–5_ and **(B)** mutant proteins R133C at 25°C and 105°C. Continuous CD spectrum measurements of **(C)** wildtype N3 EGF_1–5_ and **(D)** R133C mutant proteins with progressively increased temperature from 25°C to 125°C with 10°C as an interval.

To gain more insight into structural details, CD spectra were recorded for both WT N3 EGF_1–5_ and the R133C mutant. At room temperature (25°C), the WT N3 EGF_1–5_ and R133C recombinant proteins showed similar random coil-like structures (Figure 3A), which are in line with the predictions of tertiary structure (Figure 2A). Interestingly, at higher temperature (105°C), the two different recombinant proteins adopted a more structured conformation, as indicated by a weak negative peak at around 222 nm (Figure 3B), suggesting a structural transformation for both WT N3 EGF_1–5_ and R133C recombinant proteins. However, at high temperature, the R133C mutant is slightly more unstructured than the WT N3 EGF_1–5_ indicated by the peak at 202 nm (Figure 3B). We also investigated how the structure was transformed by slowly and progressively increasing the testing temperature from 25°C to 125°C. We observed that the structure was altered progressively as the temperature increased and that both WT N3 EGF_1–5_ and the R133C recombinant proteins showed a similar transformation profile (Figures 3C,D).

### Aggregation of WT N3 EGF_1–5_ and the R133C Mutant With and Without BRICHOS

**FIGURE 4:**
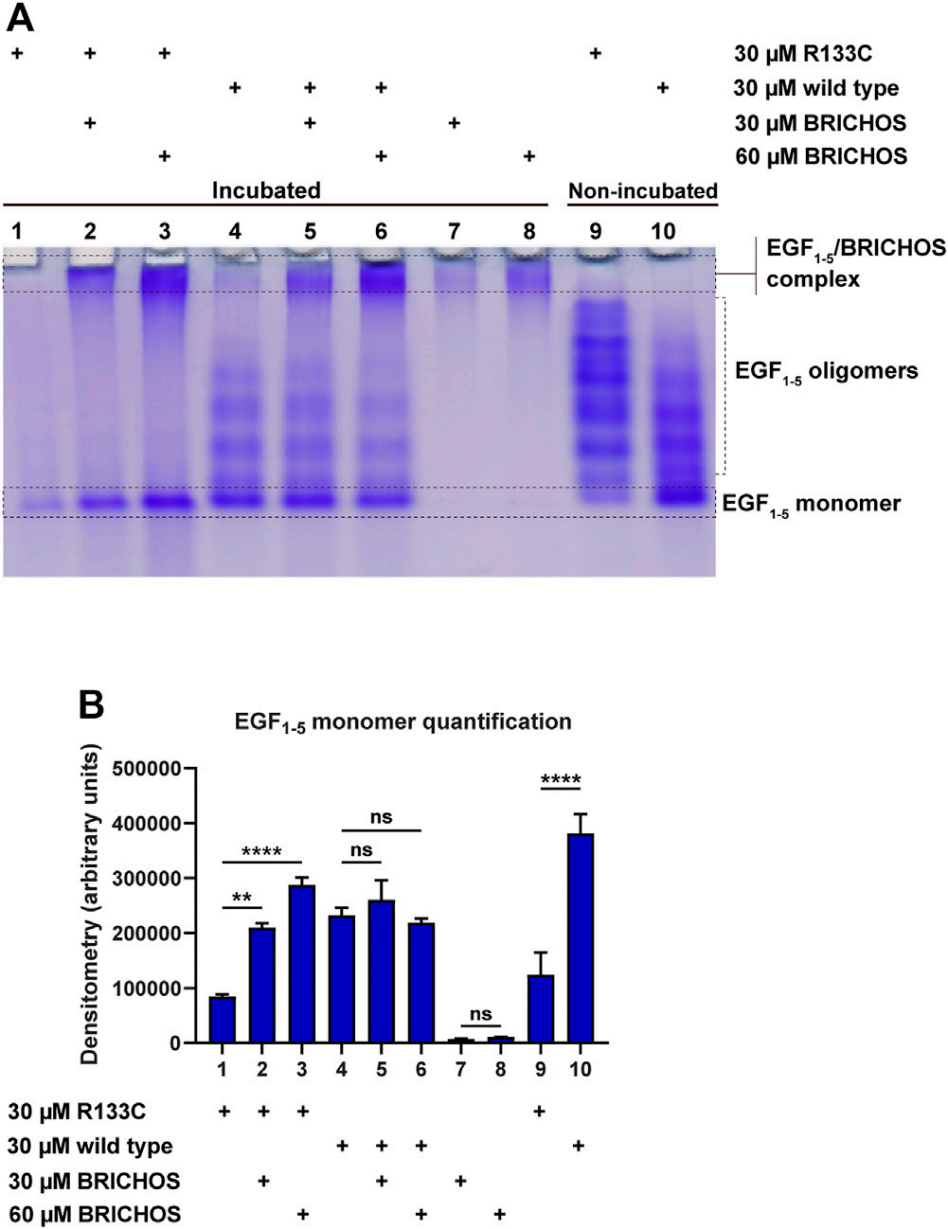
Native SDS-PAGE analysis of purified WT and R133C mutant N3 EGF_1–5_ proteins with and without Bri2 BRICHOS incubation. **(A)** The purified wildtype N3 EGF_1–5_ and R133C mutant proteins were incubated with and without Bri2 BRICHOS at 37°C for 80 h, and the final products were analyzed by native PAGE. The native SDS-PAGE is a representative image of three independent experiments. **(B)** The monomers of the EGF_1–5_ protein were quantified from three independent gels using Image Studio. The densitometry values are represented as arbitrary units, and error bars represent SD. Statistical analysis was evaluated by one-way analysis of variance (ANOVA) followed by ordinary one-way ANOVA and followed by Bonferroni's multiple comparisons test. *p* < 0.05 was considered significant (*p < 0.05, **p < 0.01, ***p < 0.001, ****p < 0.0001).

To test the aggregation properties of WT N3 EGF_1–5_ and R133C, *in vitro*, we incubated the recombinant proteins at 37°C for around 80 h with and without the Bri2 BRICHOS molecular chaperone (molar ratio 1:1 and 1:2); the final products were analyzed by native PAGE, and the monomers were quantified with Image Studio (Figures 4A,B). The mutant N3 EGF_1–5_ R133C was not visible to a large extent compared to the fresh sample without prior incubation (compare lanes 1 and 9), indicating that most of the R133C proteins were aggregating quickly. However, the WT N3 EGF_1–5_ did not aggregate after incubation as judged by the band intensities between the incubated and non-incubated fresh samples (lanes 4 and 10). When incubating mutant proteins with the molecular chaperone BRICHOS, there were more monomeric soluble N3 EGF_1–5_ R133C monomeric proteins (lane 1 compared to lanes 2 and 3), indicating that BRICHOS can stabilize N3 EGF_1–5_ mutant proteins. This effect occurs in a dose-dependent manner, as BRICHOS at a 2:1 molar ratio seems to stabilize the N3 EGF_1–5_ mutant proteins in a soluble conformation better than the 1:1 molar ratio (compare lane 3 with lane 2). Interestingly, with the Bri2 BRICHOS molecular chaperone, the oligomer states of the EGF_1–5_ R133C proteins disappeared. On the contrary, an increase in high molecular N3 EGF_1–5_/BRICHOS complexes was formed, as indicated by the significant difference in band intensity difference of the large oligomers/complex (comparing lane 2 with lane 7 and lane 3 with lane 8). In the WT, there were no apparent differences after incubation with BRICHOS. However, with BRICHOS at 2:1 ratio, the oligomer forms decreased, followed by a modest increase of the high molecular complex (compare lanes 6 and 8), indicating possible complex formation.

### Aggregation Kinetics of WT N3 EGF_1–5_ and Mutant R133C With and Without BRICHOS

**FIGURE 5:**
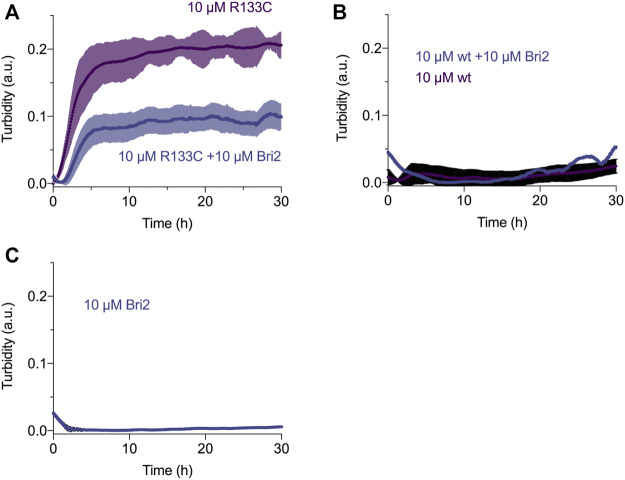
Aggregation kinetics of wildtype and R133C mutant N3 EGF_1–5_ proteins. **(A)** Aggregation kinetics of R133C mutant N3 EGF_1–5_ in the presence/absence of Bri2 BRICHOS at 1:1 ratio. **(B)** Aggregation kinetics of wildtype N3 EGF_1–5_ in the presence/absence of Bri2 BRICHOS at 1:1 ratio. **(C)** Monitoring of the aggregate of Bri2 BRICHOS alone. The shadow in each condition represents the standard error of the mean (SEM) of three independent experiments performed in triplicate (*n* = 3).

To monitor the aggregation kinetics, we recorded the heat-induced aggregation by turbidity at 360 nm at 37°C. We used a plate reader to monitor the aggregation of WT N3 EGF_1–5_ and the R133C mutant *in vitro* with and without BRICHOS. Interestingly, the recombinant R133C mutant aggregated quickly with a half time of 2.5 h (Figure 5A), while in the testing time range, the WT did not show a positive signal (Figure 5B). With the presence of BRICHOS (1:1 molar ratio), the final aggregation intensity of the R133C proteins was significantly reduced, up to 50%, in line with the native PAGE analysis. The BRICHOS protein itself did not show any obvious aggregation (Figure 5C).

### Transmission Electron Microscopy Imaging of WT N3 EGF_1–5_ and the R133C Mutant in the Presence or Absence of BRICHOS

**FIGURE 6:**
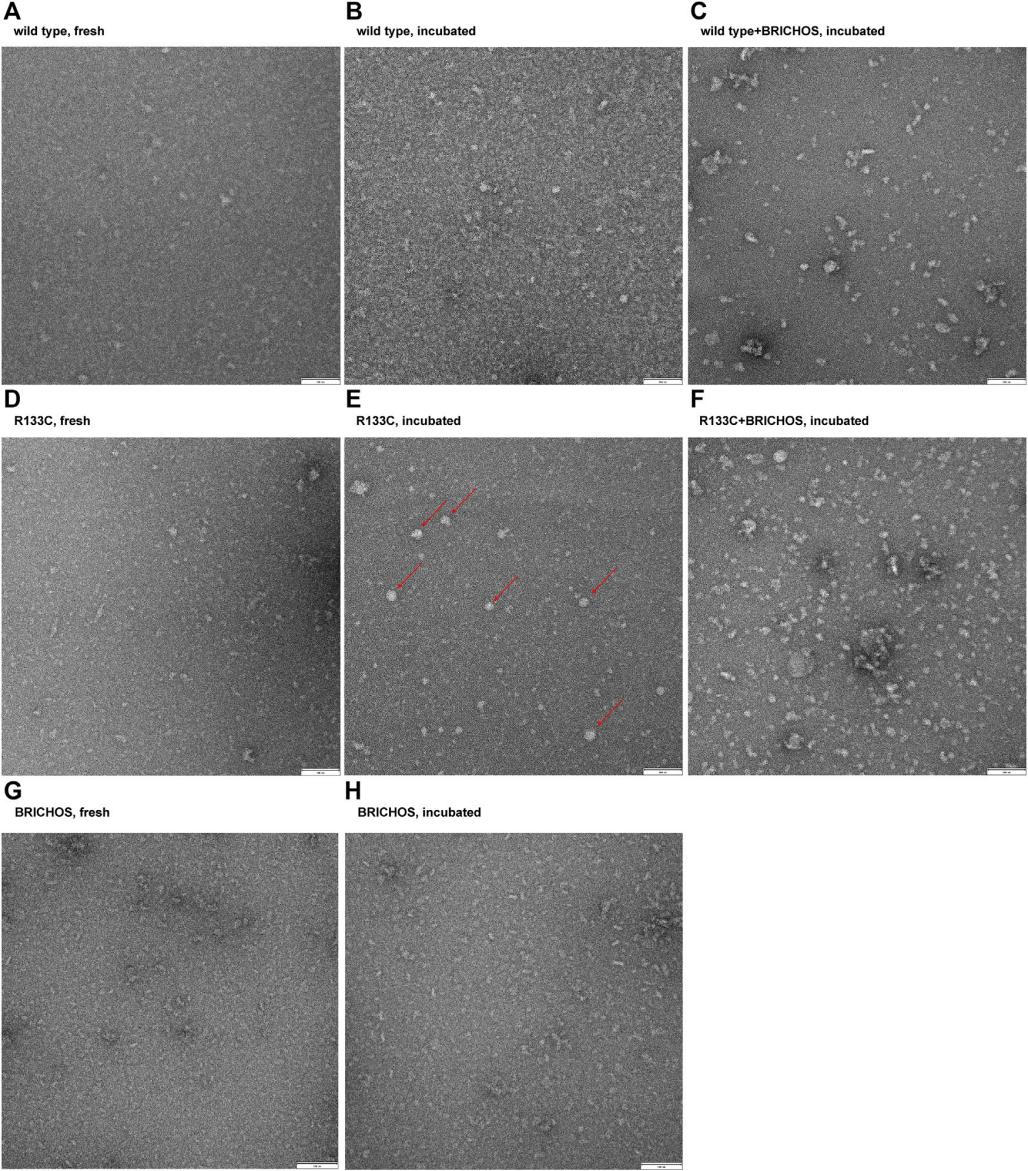
Imaging of protein particles of wildtype N3 EGF_1–5_ and R133C mutant before and after 80 h of incubation in the presence or absence of Bri2 BRICHOS. The wildtype N3 EGF_1–5_ and R133C mutant proteins were applied on carbon-coated copper grids and analyzed with TEM. **(A)** Fresh wildtype N3 EGF_1–5_. **(B)** Incubated wildtype N3 EGF_1–5_. **(C)** Incubated wildtype N3 EGF_1–5_ with Bri2 BRICHOS (1:1 ratio). **(D)** Fresh N3 EGF_1–5_ R133C mutant. **(E)** Incubated N3 EGF_1–5_ R133C mutant. **(F)** Incubated N3 EGF_1–5_ R133C mutant with Bri2 BRICHOS (1:1 ratio). **(G)** Fresh Bri2 BRICHOS. **(H)** Incubated Bri2 BRICHOS. The red arrows in F point to the large solid ball-shaped insoluble aggregates. The TEM image is a representative image of three independent experiments performed in triplicate (*n* = 3).

To observe the final products of WT N3 EGF_1–5_ and R133C before and after incubation, we imaged the protein particles by transmission electron microscopy (TEM). Before incubation, both WT N3 EGF_1–5_ and R133C showed heterogeneous but small particles (Figures 6A, D), indicating that both recombinant proteins do not aggregate at the starting point. After incubation with shaking at 37°C, the WT N3 EGF_1–5_ did not show significant changes (Figure 6B) but with slightly larger particles. On the contrary, for the R133C mutant, an increased number of larger aggregated particles was observed (Figure 6E), suggesting that the R133C mutant can facilitate the aggregation of N3 EGF_1–5_. With BRICHOS, the WT N3 EGF_1–5_ formed larger aggregates than the WT N3 EGF_1–5_ alone (Figures 6B, C), which may indicate that BRICHOS and WT N3 EGF_1–5_ form a complex. Similarly, in the presence of BRICHOS, the R133C mutant showed smaller particles compared to the mutant alone but larger than fresh samples prior to incubation (Figures 6D–F), indicating that the BRICHOS domain can stabilize the mutant N3 EGF_1–5_ R133C. No larger particles were visible with BRICHOS protein alone before and after incubation (Figures 6G, H), suggesting that the somewhat bigger particles in Figure 6F originates from the R133C mutant protein.

## Discussion

In patients with CADASIL, accumulation and aggregation of Notch3 ECD have been shown to be the main component of the pathological structure GOM surrounding the VSMCs in small arteries and capillaries, which eventually degenerate. GOMs also contain other proteins, and the hypothesis is that Notch3 ECD is recruiting and sequestering these proteins. *In vitro*, Notch3 has been shown to interact with other Notch3 proteins in a homophilic manner and that both WT and CADASIL mutant Notch3 are present in these aggregates (Opherk et al., 2009; Duering et al., 2011). However, using purified secreted Notch3 fragments (N3 EGF_1–5_) and analyzing Notch3 aggregation with a single particle approach, the authors showed that spontaneous multimerization was only limited to a CADASIL mutant Notch3 and not WT Notch3. Moreover, both forms could be detected in the aggregates using a mixture of WT and mutant N3 EGF_1–5_ (Duering et al., 2011). This suggests that the mutant Notch3 protein initiates accumulation and aggregation, but later in disease progression it acts as a seed to sequester WT Notch3 and other proteins in the extracellular matrix to form the GOM. The sequestered protein might also be a cause for the pathology due to reduced levels and disturbed homeostasis. Whether Notch signaling is affected or not is still unclear since most mutations have been shown to have neomorphic effects (neither loss nor gain of function), except for some mutations in the ligand-binding domain (Joutel et al., 2004; Peters et al., 2004; Monet et al., 2007; Monet-Lepretre et al., 2009; Arboleda-Velasquez et al., 2011). However, it cannot be ruled out that Notch3 signaling may be part of the pathological process in the later stages of the disease.

Similarly to a previous report by Duering et al., we observed that Notch3 multimerization is inhibited under reducing conditions and that both WT and R133C mutant Notch3 are present in a monomeric state (Duering et al., 2011). During non-reducing conditions, we, however, found that both WT and R133C proteins displayed protein fragments larger than the monomeric forms but slightly different sizes between the two proteins. Our interpretation is that intramolecular disulfide bonds occur within both types of proteins but that the Arg to Cys change leads to different types of disulfide interactions. Since SDS is present in both conditions, we do not observe the full spectrum of oligomers as we observe under native conditions, but at least no disulfide bond-dependent oligomers formed (Figure 1C).

The complete structure of Notch3 is not yet available. However, based on the crystal structure of parts of Notch1, we have predicted the tertiary structure of Notch3. The β-hairpin structure seems to be present in all five repeats of EGF, but most of the protein is largely unstructured. The CD spectra analysis did not reveal any differences between the WT Notch3 and the recombinant proteins of the R133C mutant. However, both proteins adopted a more structured conformation with increasing temperature in a similar progression manner. This result might be that the secondary structure is not affected by the mutation but rather the tertiary and quarternary structure due to intermolecular interactions with other proteins. This can be revealed, for example, by electron microscopy of the proteins prior to and after aggregation, since we observed that the proteins behaved differently on a native gel before and after 80 h of incubation at 37°C. The mutant protein was rapidly aggregating after incubation compared to the WT protein. In the presence of the Bri2-BRICHOS recombinant protein at a 1:1 and 1:2 molar ratio, the aggregation of the mutant protein was inhibited, as indicated by an increase in the monomeric form and a decrease in the oligomeric forms. Thus, it seems that the BRICHOS protein is stabilizing the mutant Notch3 protein in a monomeric form. The presence of high molecular weight complexes could be the composition of the Notch3 EGF_1–5_ and Bri2 BRICHOS proteins, which we also observe with transmission electron microscopy and native PAGE, where the larger particles from the incubated R133C mutant are reduced in size in the presence of Bri2-BRICHOS but are still larger than fresh non-incubated R133C-containing particles. We also confirmed that the mutant Notch3 protein aggregated faster than the WT using a turbidity assay. Aggregation was reduced by 50% in the presence of BRICHOS in a 1:1 molar ratio. In conclusion, this suggests that Bri2 BRICHOS could act as an anti-CADASIL-mutated Notch3 aggregating protein. Since accumulation of N3 ECD is an early step in the pathogenic process, followed by formation of GOM deposits and the subsequent degeneration of the VSMC, disruption of N3 misfolding and aggregation is therefore a promising and interesting therapeutic avenue to explore. Our results encourage further analysis whether other Notch3 proteins harboring different CADASIL mutations can be inhibited to aggregate by BRICHOS, which will be of particular importance for the clinical translatability of a therapy for CADASIL.

## Data Availability

The raw data supporting the conclusion of this article will be made available by the authors, without undue reservation.

## References

[B1] Andersson E. R. Sandberg R. Lendahl U. (2011). Notch Signaling: Simplicity in Design, Versatility in Function. Development. 138 (17), 3593–3612. 10.1242/dev.063610 21828089

[B2] Arboleda-Velasquez J. F. Manent J. Lee J. H. Tikka S. Ospina C. Vanderburg C. R. (2011). Hypomorphic Notch 3 Alleles Link Notch Signaling to Ischemic Cerebral Small-Vessel Disease. Proc. Natl. Acad. Sci. 108 (21), E128–E135. 10.1073/pnas.1101964108 21555590 PMC3102344

[B3] Balchin D. Hayer-Hartl M. Hartl F. U. (2016). *In Vivo* aspects of Protein Folding and Quality Control. Science. 353 (6294), aac4354. 10.1126/science.aac4354 27365453

[B4] Chabriat H. Joutel A. Dichgans M. Tournier-Lasserve E. Bousser M.-G. (2009). Cadasil. Lancet Neurol. 8 (7), 643–653. 10.1016/S1474-4422(09)70127-9 19539236

[B5] Chen G. Abelein A. Nilsson H. E. Leppert A. Tambaro S. (2017). Bri2 BRICHOS Client Specificity and Chaperone Activity Are Governed by Assembly State. Nat. Commun. 8 (1), 2081. 10.1038/s41467-017-02056-4 29234026 PMC5727130

[B6] Domenga V. Fardoux P. Lacombe P. Monet M. Maciazek J. Krebs L. T. (2004). Notch3 Is Required for Arterial Identity and Maturation of Vascular Smooth Muscle Cells. Genes Dev. 18 (22), 2730–2735. 10.1101/gad.308904 15545631 PMC528893

[B7] Duering M. Karpinska A. Rosner S. Hopfner F. Zechmeister M. Peters N. (2011). Co-Aggregate Formation of CADASIL-Mutant NOTCH3: a Single-Particle Analysis. Hum. Mol. Genet. 20 (16), 3256–3265. 10.1093/hmg/ddr237 21628316

[B8] Hedlund J. Johansson J. Persson B. (2009). BRICHOS - a Superfamily of Multidomain Proteins with Diverse Functions. BMC Res. Notes. 2, 180. 10.1186/1756-0500-2-180 19747390 PMC2751770

[B9] Joutel A. Andreux F. Gaulis S. Domenga V. Cecillon M. Battail N. (2000). The Ectodomain of the Notch3 Receptor Accumulates within the Cerebrovasculature of CADASIL Patients. J. Clin. Invest. 105 (5), 597–605. 10.1172/JCI8047 10712431 PMC289174

[B10] Joutel A. Corpechot C. Ducros A. Vahedi K. Chabriat H. Mouton P. (1996). Notch3 Mutations in CADASIL, a Hereditary Adult-Onset Condition Causing Stroke and Dementia. Nature. 383 (6602), 707–710. 10.1038/383707a0 8878478

[B11] Joutel A. Favrole P. Labauge P. Chabriat H. Lescoat C. Andreux F. (2001). Skin Biopsy Immunostaining with a Notch3 Monoclonal Antibody for CADASIL Diagnosis. The Lancet. 358 (9298), 2049–2051. 10.1016/S0140-6736(01)07142-2 11755616

[B12] Joutel A. Monet M. Domenga V. Riant F. Tournier-Lasserve E. (2004). Pathogenic Mutations Associated with Cerebral Autosomal Dominant Arteriopathy with Subcortical Infarcts and Leukoencephalopathy Differently Affect Jagged1 Binding and Notch3 Activity via the RBP/JK Signaling Pathway. Am. J. Hum. Genet. 74 (2), 338–347. 10.1086/381506 14714274 PMC1181931

[B13] Joutel A. Monet-Leprêtre M. Gosele C. Baron-Menguy C. Hammes A. Schmidt S. (2010). Cerebrovascular Dysfunction and Microcirculation Rarefaction Precede White Matter Lesions in a Mouse Genetic Model of Cerebral Ischemic Small Vessel Disease. J. Clin. Invest. 120 (2), 433–445. 10.1172/JCI39733 20071773 PMC2810078

[B14] Krebs L. T. Xue Y. Norton C. R. Sundberg J. P. Beatus P. Lendahl U. (2003). Characterization ofNotch3-Deficient Mice: Normal Embryonic Development and Absence of Genetic Interactions with aNotch1 Mutation. Genesis. 37 (3), 139–143. 10.1002/gene.10241 14595837

[B15] Luca V. C. Kim B. C. Ge C. Kakuda S. Wu D. Roein-Peikar M. (2017). Notch-Jagged Complex Structure Implicates a Catch Bond in Tuning Ligand Sensitivity. Science. 355 (6331), 1320–1324. 10.1126/science.aaf9739 28254785 PMC5459593

[B16] Monet M. Domenga V. Lemaire B. Souilhol C. Langa F. Babinet C. (2007). The Archetypal R90C CADASIL-NOTCH3 Mutation Retains NOTCH3 Function *In Vivo* . Hum. Mol. Genet. 16 (8), 982–992. 10.1093/hmg/ddm042 17331978

[B17] Monet-Leprêtre M. Bardot B. Lemaire B. Domenga V. Godin O. Dichgans M. (2009). Distinct Phenotypic and Functional Features of CADASIL Mutations in the Notch3 Ligand Binding Domain. Brain. 132 (Pt 6), 1601–1612. 10.1093/brain/awp049 19293235 PMC2685919

[B18] Monet-Leprêtre M. Haddad I. Baron-Menguy C. Fouillot-Panchal M. Riani M. Domenga-Denier V. (2013). Abnormal Recruitment of Extracellular Matrix Proteins by Excess Notch3ECD: a New Pathomechanism in CADASIL. Brain. 136 (Pt 6), 1830–1845. 10.1093/brain/awt092 23649698 PMC3673461

[B19] Moreton F. C. Razvi S. S. M. Davidson R. Muir K. W. (2014). Changing Clinical Patterns and Increasing Prevalence in CADASIL. Acta Neurol. Scand. 130 (3), 197–203. 10.1111/ane.12266 24840674

[B20] Narayan S. K. Gorman G. Kalaria R. N. Ford G. A. Chinnery P. F. (2012). The Minimum Prevalence of CADASIL in Northeast England. Neurology. 78 (13), 1025–1027. 10.1212/WNL.0b013e31824d586c 22422895 PMC3310314

[B21] Opherk C. Duering M. Peters N. Karpinska A. Rosner S. Schneider E. (2009). CADASIL Mutations Enhance Spontaneous Multimerization of NOTCH3. Hum. Mol. Genet. 18 (15), 2761–2767. 10.1093/hmg/ddp211 19417009

[B22] Opherk C. Peters N. Herzog J. Luedtke R. Dichgans M. (2004). Long-term Prognosis and Causes of Death in CADASIL: a Retrospective Study in 411 Patients. Brain. 127 (Pt 11), 2533–2539. 10.1093/brain/awh282 15364702

[B23] Pantoni L. (2010). Cerebral Small Vessel Disease: from Pathogenesis and Clinical Characteristics to Therapeutic Challenges. Lancet Neurol. 9 (7), 689–701. 10.1016/S1474-4422(10)70104-6 20610345

[B24] Peters N. Opherk C. Zacherle S. Capell A. Gempel P. Dichgans M. (2004). CADASIL-associated Notch3 Mutations Have Differential Effects Both on Ligand Binding and Ligand-Induced Notch3 Receptor Signaling Through RBP-Jk. Exp. Cell Res. 299 (2), 454–464. 10.1016/j.yexcr.2004.06.004 15350543

[B25] Pettersen E. F. Goddard T. D. Huang C. C. Couch G. S. Greenblatt D. M. Meng E. C. (2004). UCSF Chimera?A Visualization System for Exploratory Research and Analysis. J. Comput. Chem. 25 (13), 1605–1612. 10.1002/jcc.20084 15264254

[B26] Pfefferkorn T. von Stuckrad-Barre S. Herzog J. Gasser T. Hamann G. F. Dichgans M. (2001). Reduced Cerebrovascular CO 2 Reactivity in CADASIL. Stroke. 32 (1), 17–21. 10.1161/01.str.32.1.17 11136908

[B27] Poska H. Haslbeck M. Kurudenkandy F. R. Hermansson E. Chen G. Kostallas G. (2016). Dementia-related Bri2 BRICHOS Is a Versatile Molecular Chaperone that Efficiently Inhibits Aβ42 Toxicity in Drosophila. Biochem. J. 473 (20), 3683–3704. 10.1042/BCJ20160277 27514716

[B28] Razvi S. S. M. Davidson R. Bone I. Muir K. W. (2005). Is Inadequate Family History a Barrier to Diagnosis in CADASIL? Acta Neurol. Scand. 112 (5), 323–326. 10.1111/j.1600-0404.2005.00495.x 16218915

[B29] Roy A. Kucukural A. Zhang Y. (2010). I-TASSER: a Unified Platform for Automated Protein Structure and Function Prediction. Nat. Protoc. 5 (4), 725–738. 10.1038/nprot.2010.5 20360767 PMC2849174

[B30] Ruchoux M. M. Guerouaou D. Vandenhaute B. Pruvo J.-P. Vermersch P. Leys D. (1995). Systemic Vascular Smooth Muscle Cell Impairment in Cerebral Autosomal Dominant Arteriopathy with Subcortical Infarcts and Leukoencephalopathy. Acta Neuropathol. 89 (6), 500–512. 10.1007/bf00571504 7676806

[B31] Rutten J. W. Dauwerse H. G. Gravesteijn G. Belzen M. J. Grond J. Polke J. M. (2016). Archetypal NOTCH3 Mutations Frequent in Public Exome: Implications for CADASIL. Ann. Clin. Transl Neurol. 3 (11), 844–853. 10.1002/acn3.344 27844030 PMC5099530

[B32] Rutten J. W. Haan J. Terwindt G. M. van Duinen S. G. Boon E. M. Lesnik Oberstein S. A. (2014). Interpretation ofNOTCH3mutations in the Diagnosis of CADASIL. Expert Rev. Mol. Diagn. 14 (5), 593–603. 10.1586/14737159.2014.922880 24844136

[B33] Sánchez-Pulido L. Devos D. Valencia A. (2002). BRICHOS: a Conserved Domain in Proteins Associated with Dementia, Respiratory Distress and Cancer. Trends Biochem. Sci. 27 (7), 329–332. 10.1016/s0968-0004(02)02134-5 12114016

